# Transperineal MRI-US Fusion-Guided Target Biopsy of the Prostate after Abdominoperineal Resection: A Case Report

**DOI:** 10.5334/jbsr.2583

**Published:** 2021-10-14

**Authors:** Nando De Vulder, Koen Geldof, Frederic Baekelandt, Katrien Gieraerts

**Affiliations:** 1 AZ Sint-Jan Brugge, BE; 2 AZ Sint-Lucas Brugge, BE

**Keywords:** Abdominoperineal Resection, Prostate Cancer, Target Biopsy, MRI-US Fusion, Case Reports

## Abstract

**Main Teaching Point**: MRI-US fusion-guided transperineal prostate biopsy can help sampling clinically relevant prostate cancer in patients with a history of abdominoperineal resection and is feasible under local anaesthesia and in an outpatient setting.

Prostate cancer remains one of the most diagnosed cancers in men worldwide. Prostate biopsy in patients with a history of abdominoperineal resection poses a serious challenge since transrectal ultrasound is not possible. Several techniques have been described with their respective limitations and risks. This report of such a patient aged 75, presents a Magnetic Resonance Imaging-Ultrasound fusion-guided transperineal approach to prostate biopsy which can be performed under local anaesthesia and in an outpatient setting.

## Introduction

Prostate cancer (PCa) is still one of the most diagnosed cancers in men worldwide [1,2]. Diagnosis is usually based on physical examination with digital rectal examination (DRE) and/or elevated serum prostate specific antigen (PSA). The work-up includes multiparametric magnetic resonance imaging (mpMRI), which has been shown to have good sensitivity for detecting and localizing prostate cancer [3]. However, definitive diagnosis requires histopathology. The latest guidelines of the European Association of Urology (EAU) on PCa propose the transperineal approach as the standard of care [1]. Screening and diagnosis of PCa in men without an anal canal presents the clinician with several difficulties, since DRE and transrectal ultrasound (TRUS) are not possible. Biopsy also poses a particular challenge. Approaches via the transperineal or transgluteal route and also transabdominal biopsies have been described, guided by (transperineal, transabdominal or transurethral) ultrasound (US), computed tomography (CT) or MRI [3,4]. We present an MRI-US fusion-guided transperineal targeted approach to prostate biopsy in a patient with prior abdomino-perineal resection, in an outpatient setting under local anaesthesia.

## Case Report

**Figure 1:**
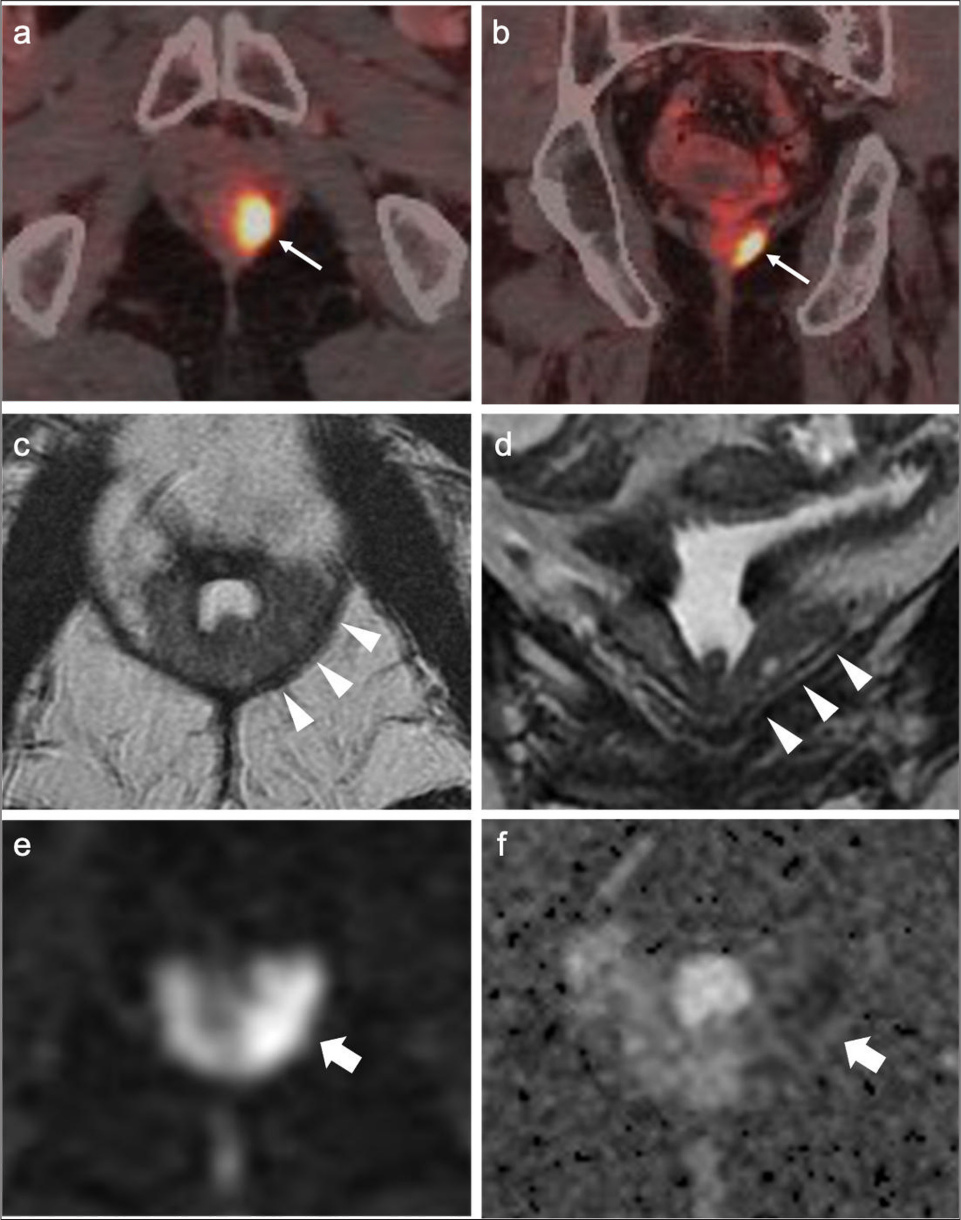
**(a, b)** Transverse and Coronal PSMA-PET/CT images. A small prostate after TURP is seen with tracer capture (thin arrows) in the left prostate lodge, suspected for locoregional recurrence; **(c, d)** Transverse and Coronal T2 weighted MR images. These images demonstrate a small gland of only 8.5 cc secondary to TURP and show a diffuse T2 hypointensity of the peripheral zone. On the left, there is a diffuse, more pronounced thickening of the peripheral zone with mainly subcapsular disruption of the internal architecture in the centre, extending from the apex to the posterolateral base of the prostate (arrowheads); **(e)** Diffusion Weighted Imaging of the prostate. A high signal intensity is seen, indicating restricted diffusion in the left peripheral zone (thick arrow); **(f)** ADC map of the prostate. ADC values of 660–710 confirm an unequivocal restricted diffusion in the left peripheral zone (thick arrow), suggesting high-grade malignancy.

A 75-year-old man presented to the urology department with progressive PSA elevation. The patient had a history of rectal adenocarcinoma treated with neoadjuvant radiotherapy and Abdomino-Perineal Resection (APR), which precluded DRE and TRUS. The patient had already undergone a transurethral resection of the prostate (TURP) and CT-guided prostate biopsy twice, but no prostate cancer was identified despite a progressively rising PSA. Prostate Specific Membrane Antigen – Positron Emission Tomography/CT (PSMA-PET/CT) showed suspect local tracer capture (***Figure 1a*** and ***1b***). Multiparametric MRI demonstrated a small gland of only 8.5 cc secondary to TURP and showed a diffuse T2 hypointensity of the peripheral zone, with a more pronounced thickening and mainly subcapsular disruption of the internal architecture on the left (***Figure 1c*** and ***1d***). The perfusion study showed hypervascular contrast enhancement and the entire zone showed an unequivocal restricted diffusion suggesting high-grade malignancy (***Figure 1e*** and ***1f***). The periphery was scored according to the Prostate Imaging-Reporting and Data System v2: PI-RADS 5. Further management preferred a third attempt at prostate biopsy given the high probability of malignancy. MRI-US fusion-guided target biopsies of the prostate with transperineal ultrasound were performed under local anaesthesia, in an outpatient setting and without antibiotic prophylaxis (as the EAU guidelines had not yet been published at the time of the biopsy).

### Technique

**Figure 2:**
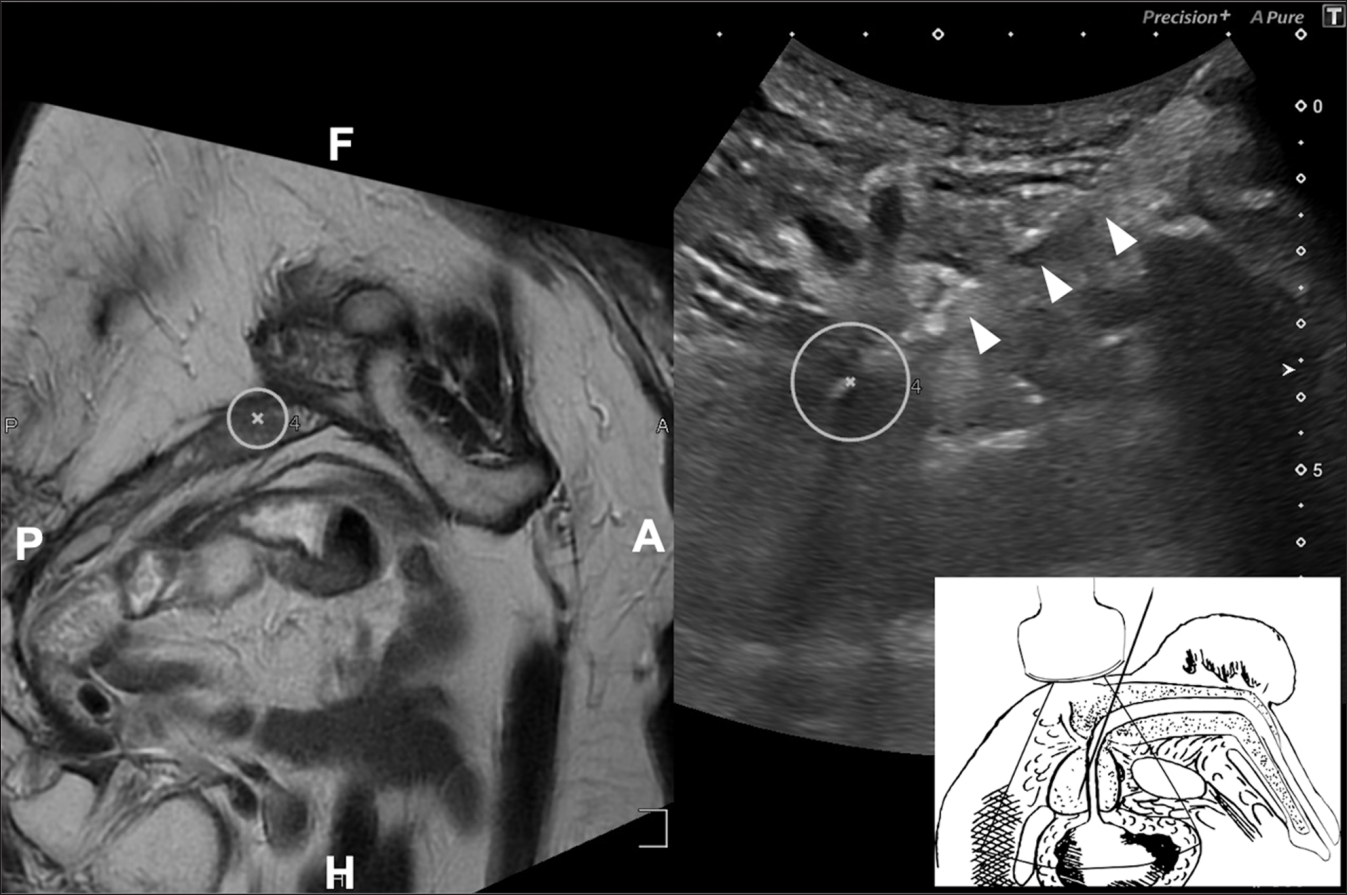
Sagittal MRI (left) and transperineal US fusion (right) as seen on the ultrasound system monitor during biopsy. The biopsy needle (arrows) enters the ROI (circle) during sampling of the PI-RADS 5 lesion in the small prostate after TURP. Despite the inferior quality of transperineal US compared to transrectal ultrasound when imaging the prostate, software fusion allows accurate sampling. In the right lower corner, drawing of transperineal prostate biopsy. The convex US probe is placed sagitally on the patient’s perineum, introducing the biopsy needle anteriorly of the probe. Adapted with permission from Shinohara et al. [4]. A = Anterior; P = Posterior; F = Feet; H = Head.

The Aplio i800 ultrasound system (Canon Medical Systems, Japan), equipped with specialised fusion software, was used. The patient was placed in the lithotomy position with the scrotum anteriorly retracted and the perineal skin was thoroughly disinfected with betadine solution. Local anaesthetics were applied starting with superficial anaesthesia of the perineal skin with infiltration of 10 ml linisol 1 % in a wide area. Deep periprostatic nerve blockade consisted of 20 ml linisol 1% combined with 10 ml Ropivacain and was administered bilaterally in two injections. Ultrasound scanning was performed with the curved Ultra-Wideband Multi-Frequency iDMS Convex (i8CX1) ultrasound probe (Canon Medical Systems, Japan). MRI images were pre-imported into the ultrasound system. A region of interest (ROI) was added to MRI on the ultrasound system to indicate the area suspected of being malignant and guide the targeted biopsy. The absence of the anal canal necessitated a transabdominal ultrasound approach to start the MRI-US fusion and therefore the images were manually fused using anatomical landmarks. The cranial and caudal most aspects of the pubic symphysis were the primary fusion points to create a rigid overlay of the axially oriented transabdominal ultrasound images over the axial MRI. Next, the ultrasound probe was placed sagittally on the perineum and some further minor adjustments were made to optimise the image overlay and thus the MRI-US correlation (***Figure 2***). Biopsies were taken freehand using the Magnum™ reusable core biopsy instrument with the disposable 18 Gauge TruGuide™ coaxial biopsy needle (Bard, Covington, GA, USA). During sample collection, the ultrasound probe was placed sagittally on the patient’s perineum to visualise the prostate while introducing the needle anteriorly towards the suspected lesion. Due to the small prostate volume, no systematic biopsies were performed.

### Results

Six transperineal MRI-US fusion-guided target biopsy cores were taken from the left peripheral zone. The maximum size of the cores was 1.5 cm and all six cores contained adenocarcinoma foci. The largest focus measured 7 mm with a Gleason score of 4 + 4 = 8 (Gleason grade group 4*). Up to 80% adenocarcinoma was found in one core and a total of 60% of the material contained adenocarcinoma. The patient was thus diagnosed with a locally advanced high risk prostate cancer, necessitating the initiation of androgen deprivation therapy. Local treatment of the cancer was not deemed possible due to the APR and history of perirectal radiotherapy.

## Discussion

In patients with a history of APR, transperineal biopsy guided by transabdominal ultrasound can lead to inaccuracy in visualising the prostate and difficulties in positioning the needle, and transurethral guidance is of little use for needle guidance. The transabdominal approach carries the risk of injury to the bowel or dorsal artery complex, and CT and MRI are time-consuming and expensive [2,3,4]. MRI before biopsy has been shown to increase the detection rate of prostate cancer, particularly clinically significant cancer, and cognitive MRI-US fusion techniques have been described as a feasible approach [3]. Other US fusion techniques such as PSMA-PET/US guided biopsies have also demonstrated the ability to diagnose clinically relevant PCa [5]. In this case we could have chosen to perform the fusion using the PSMA-PET/CT images, but our experience is that software fusion is easier to perform using MRI because of the higher tissue contrast and thus more accurate anatomical landmarks. Using software-based MRI-US fusion, we can localise suspicious lesions better, despite the inferior quality of transperineal ultrasound compared to transrectal ultrasound when imaging the prostate.

## Conclusion

In this report we present a feasible technique for MRI-US fusion-guided transperineal prostate biopsy in patients with a history of APR. The technique is able to detect clinically relevant prostate cancer with good quality core samples and could potentially also be used in other patient groups where the anal canal is absent or preferably avoided such as ileal pouch or distal anal anastomosis.
